# Motor Imagery Analysis from Extensive EEG Data Representations Using Convolutional Neural Networks

**DOI:** 10.3390/s22166093

**Published:** 2022-08-15

**Authors:** Vicente A. Lomelin-Ibarra, Andres E. Gutierrez-Rodriguez, Jose A. Cantoral-Ceballos

**Affiliations:** 1 Tecnologico de Monterrey, School of Engineering and Sciences, Monterrey 64849, Mexico; 2 MAHLE Shared Services, Monterrey 64650, Mexico

**Keywords:** deep learning, motor imagery, motor skill impairment

## Abstract

Motor imagery is a complex mental task that represents muscular movement without the execution of muscular action, involving cognitive processes of motor planning and sensorimotor proprioception of the body. Since the mental task has similar behavior to that of the motor execution process, it can be used to create rehabilitation routines for patients with some motor skill impairment. However, due to the nature of this mental task, its execution is complicated. Hence, the classification of these signals in scenarios such as brain–computer interface systems tends to have a poor performance. In this work, we study in depth different forms of data representation of motor imagery EEG signals for distinct CNN-based models as well as novel EEG data representations including spectrograms and multidimensional raw data. With the aid of transfer learning, we achieve results up to 93% accuracy, exceeding the current state of the art. However, although these results are strong, they entail the use of high computational resources to generate the samples, since they are based on spectrograms. Thus, we searched further for alternative forms of EEG representations, based on 1D, 2D, and 3D variations of the raw data, leading to promising results for motor imagery classification that still exceed the state of the art. Hence, in this work, we focus on exploring alternative methods to process and improve the classification of motor imagery features with few preprocessing techniques.

## 1. Introduction

Motor skill impairment (MSI) is a common symptom in certain conditions and diseases. Health conditions such as strokes, brain injuries, and neuromuscular diseases present a progressive degradation of the use of motor function and fine motor skills [1]. This condition affects the quality of life of those that suffer from a form of neurological or neuromuscular disorders. It prevents them from executing simple motor tasks such as grasping or walking, to complete motor paralysis [2]. Nevertheless, cognitive processes associated with motor execution remain intact in some of the cases. These processes make use of areas of the brain that interact directly with the cerebral cortex involved with muscle movement. This is the case for motor planning, which is in charge of planning sequential muscle action to execute specific movements [3].

The brain’s communication system is complex, consisting of the synapses and interaction of neurons to excite or inhibit each other through an electrochemical transmission of signals. Cognitive processes make use of large and specialized neural networks to receive, integrate, and transmit information [4]. The action potential of this electrical behavior can be detected through electroencephalography (EEG). EEG signal acquisition is a non-invasive method that registers the brain’s neuron’s action potential [5]. EEG 1D signals present a strong temporal domain; however, due to the dissipation of electrical currents through the scalp, the spatial domain causes trouble to isolate specific mental tasks [6,7]. Through EEG signal acquisition, a link between the brain and a computer can be established, allowing the computer to execute commands with the identification of particular patterns of neural activity [8,9]. However, due to the complex nature of the cognitive process, exhaustive subject training to dominate the mental task is required to simplify the preprocessing of the signals and to improve the classification of the extracted features [7]. Still, studies have delivered fruitful results in the rehabilitation methods employed with these systems, integrating forms of sensorial feedback to the patients, returning some mobility to affected limbs [10].

In the literature, a variety of machine learning classifier methods have been explored for MI-based EEG classification. Common methodologies make use of preprocessing to reduce artifact interference of the signals and signal processing to isolate the frequency range of specific cognitive processes [11,12,13]. This allows extracting features from EEG signals and converting them into commands for the BCI system [14]. For instance, the combination of fast Fourier transform (FFT) to extract features with quadratic linear discriminant analysis (LDA) for classification, and Naive Bayes classification. Unfortunately, without an exhaustive subject training for MI execution, the classification performance of the BCI systems tends to be poor [15]. MI has proven to be a difficult and complex mental task to master, having an average of around 70% accuracy performance on offline studies [16,17].

In recent years, the application of deep learning (DL) has stretched to analyze 1D signals that describe a variety of physiological behaviors. The literature expands into different approaches to analyze physiological data from different sources for a variety of medical applications [18]. For instance, electromyography signals are used to determine tension patterns on hand motion and different muscle activity [19], and classification of heart diseases and detection of abnormal variations of the heart’s rhythm through electrocardiography data [20]. Likewise, physiological signals obtained through EEG are also studied for the classification of different mental tasks, such as MI [18]. In order to address EEG’s low spatial resolution, a variety of methods have been employed to improve the representation of these 1D signals [21]. For instance, the transformation of the EEG signals into spectrograms allows changing the data into a 2D frequency image representation of the signal, allowing CNN models to extract features from an image-based representation [22,23]. The construction of these EEG representations has a wide variety of variations to account for the spatial distribution of the electrodes used for the raw EEG recording. For instance, ref. [24] presents an approach that combines the spectrograms of three channels (C3, Cz, and C4) into a single image to be used as input for a CNN model for a four-class classification task. Another approach for image-based MI classification is the one presented in [25], where the spectrogram images generated from each of the channels are arranged into a single image to form a topological map based on the EEG’s electrode position, which in turn is used as input for a CNN model. In the case of raw EEG signals, the study [26] presents an approach in which the 64-channel EEG recording raw signals are presented as an input for a CNN model. The model performs a 1D temporal convolution over each channel and a second spatial convolution over the channel axis, allowing us to analyze the EEG signal as a 2D non-image form of input.

**Figure 1:**
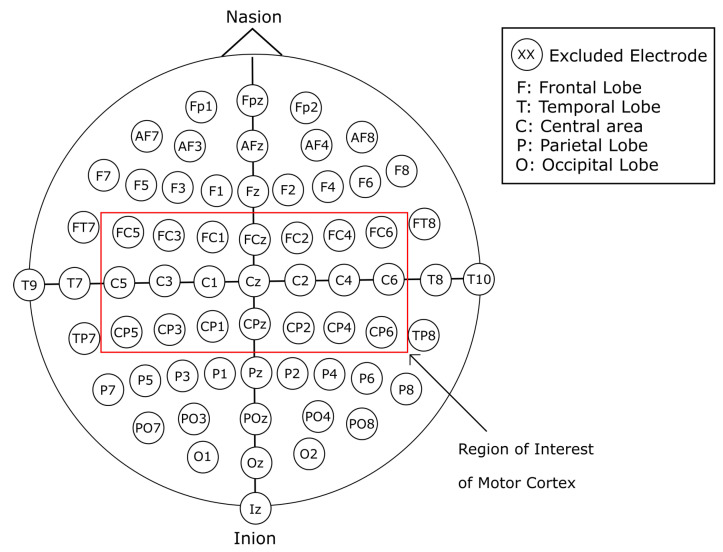
Highlight of motor cortex channels (red box) that were considered for spatial reduction.

In this work, we present a comprehensive exploration of a variety of input representations for EEG signals. These different data inputs take into consideration image-based representations of the EEG signals, as well as 1D, 2D, and 3D array representations of the raw EEG signal data. The forms of representation were designed around the motor cortex electrode positioning, as shown in Figure 1. Additionally, the data preparation was worked around channels C3, Cz, and C4, which according to literature are the channels that present features within the EEG signal that constitutes MI tasks [22,24]. We designed and implemented different CNN models with a varied number of layers, with and without transfer learning, in combination with the single and two-layered models for the image-based representation of the EEG signal. Furthermore, models with four and five layers for the 1D raw data input were designed. Hence, we can summarize our contribution as follows:

- Novel EEG signal transformations to account for spatial distributions of electrode placements.
- Novel DL models that were explicitly designed to address the strengths of each of the different EEG data representations, with results that exceed those presented previously in the state of the art.

## 2. Materials and Methods

### 2.1. Dataset Description and Preparation

**Figure 2:**
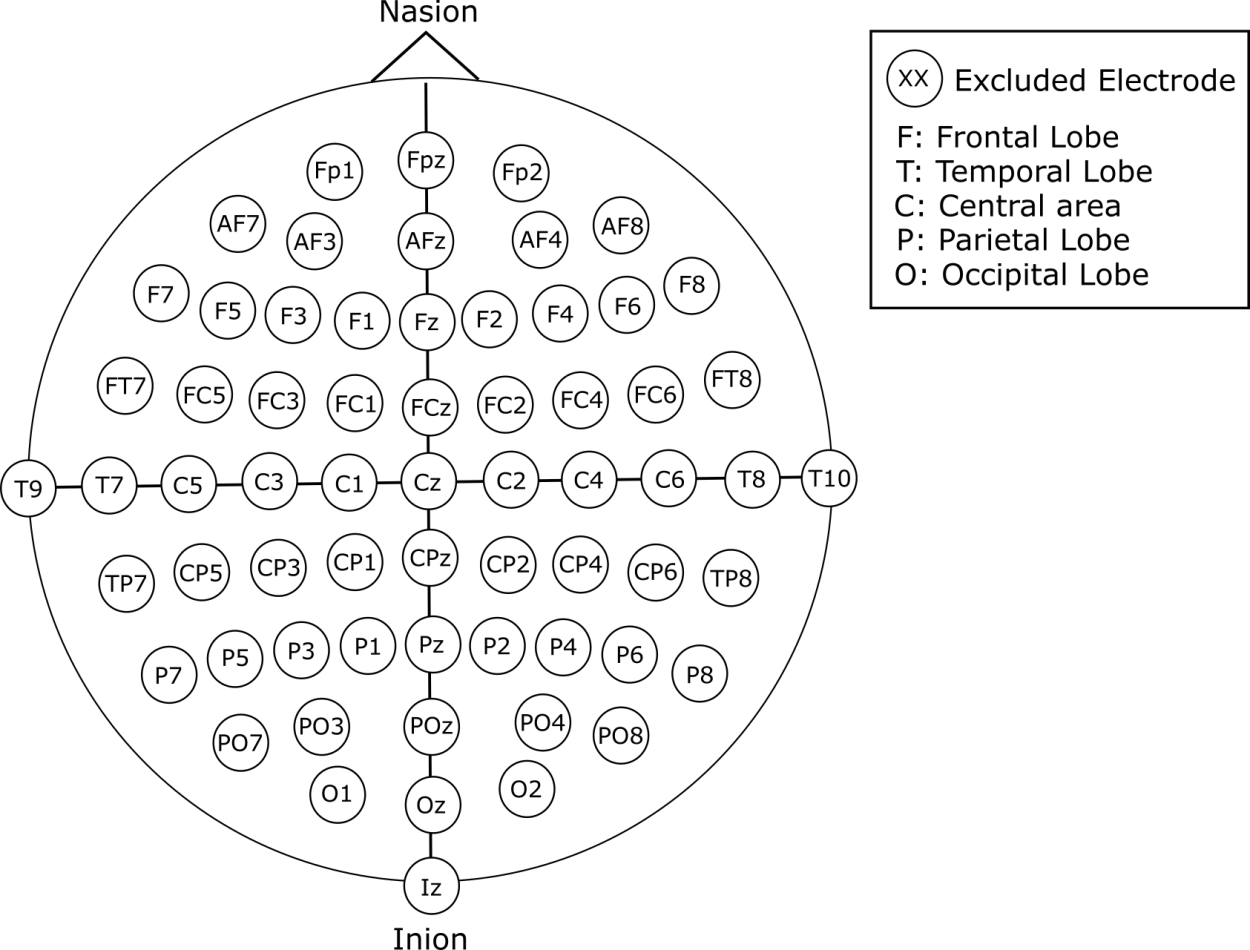
Electrode placement in accordance to the 10-10 international system. Electrodes are identified with labels. Each letter corresponds to a location of the cortex: F: frontal lobe, T: temporal lobe, C: central area O: occipital lobe, P: parietal lobe. A combination of these labels indicates intermediate areas of the cortex (PO indicates the parietal–occipital area).

**Figure 3:**
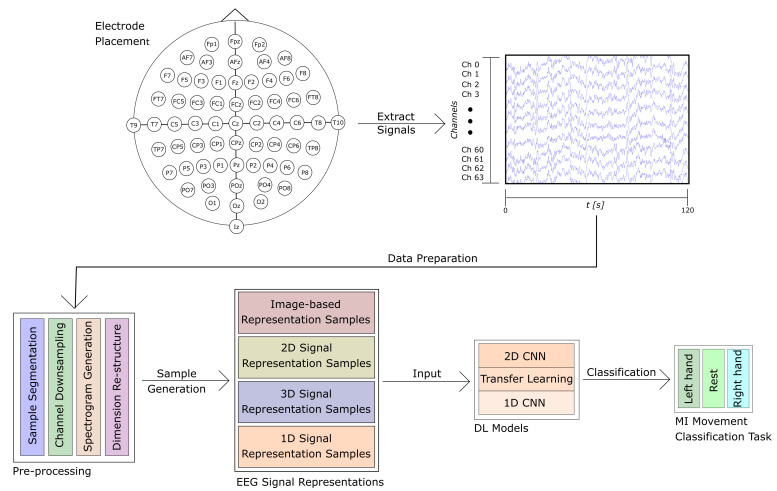
Once the signals are acquired, the recordings are segmented into four-second intervals to generate the samples for each class. Samples are prepared as described in Section 2.2 and Section 2.3, with signal transformation to spectrogram images, data dimension re-structuring, and channel downsampling. Finally, the samples are used as input for the proposed models described in Section 2.4 and Section 2.5 for the 3-class classification of MI for rest, left-, and right-hand movement.

For this work, we use the publicly available Physionet’s Physiobank of Motor Movement/Motor Imagery Database [27]. The database consists of 109 subjects, each with 14 recordings of approximately 120 s. The EEG signal recordings were acquired using an EEG with 64 channels positioned according to the 10-10 international system excluding electrodes Nz, F9, F10, FT9, FT10, A1, A2, TP9, TP10, P9, and P10, as shown in Figure 2 with a sampling frequency of 160 Hz. Out of the 14 recordings for each subject, 2 of them represent baseline recordings with open and closed eyes, respectively. The remaining 12 recordings are task-related, and contain 3 classes per recording with an approximate duration of 4 s per class, for a total of 30 samples per recording. The content of each recording is shown in Table 1. The experimental protocol consists of four tasks. The onset and duration for each class are annotated in the file, and are denominated as T0 for rest, T1 for the onset of motion (real or imagined) of the left hand or both hands, and T2 for onset of motion (real or imagined) of the left hand or both feet. The proposed process for this work is simplified in Figure 3.

The recordings of interest for this work are those from Task 2, which would correspond to motor imagery movement of either left or right hand. Therefore, we used three recordings for each subject in the database: Recording 4, Recording 8, and Recording 12. As previously mentioned, three classes were considered for this work. The three classes for this work are left-hand MI, right-hand MI, and rest. Due to incomplete annotations, the recordings from subjects 88, 92, 100, and 104 were excluded, leaving 105 subjects that were used for this work.

Alternative forms of representations were adopted for this work with the purpose of analyzing the EEG signal. The spectrogram image representation is a common method that has been employed to analyze EEG as well as other time-varying signals. These images generated from the EEG signal can represent its behavior in the frequency-time domain and differentiate the neural activity in different frequency bands and the difference in energy levels tied to the execution of a mental task. Additionally, the channel down-sampling variations allow limiting the information from the recordings to the area of interest, i.e., those closer to the motor cortex. Channel selection is a common method employed for EEG analysis. The selection is most commonly employed with a symmetrical pairing of electrodes over the region of interest from both hemispheres [28]. This work performs the channel selection process as sub-regions of interest of the motor cortex, and generate additional samples of the MI mental task execution in both hemispheres of the brain. This is supported because the execution of MI tasks has been related to specific areas of the brain, such as the prefrontal cortex, the supplementary motor area, and the motor cortex.

For all the distinct data representations, 80% of all the generated samples were randomly selected for training, and 10% were randomly selected for a validation set, with the remaining 10% of the samples belonging to the test set. This partition method is based and that presented in previous research [29] and is supported given the high intra-class time variability within the same EEG recording [3], showing differences within each subject and, thus among different subjects. These differences offer an inherent way to classify MI tasks.

### 2.2. Spectrogram Image

Spectrogram images generated from an EEG recording allow to analyze the signal in the time-frequency domain using standard computer vision techniques. Many studies have taken advantage of the information that can be obtained from this domain. For this work, the EEG signals containing Task 2 were used to generate the spectrogram images. Two methods to generate spectrogram image representations of the signals were considered. The first method generates single-channel spectrogram images, generating an image for each interval of time representing a class for each of the channels. The second method vertically stacks together the resulting spectrograms for each channel, and then transforms them into an image.

#### 2.2.1. Single EEG Channel Spectrogram Images

**Figure 4:**
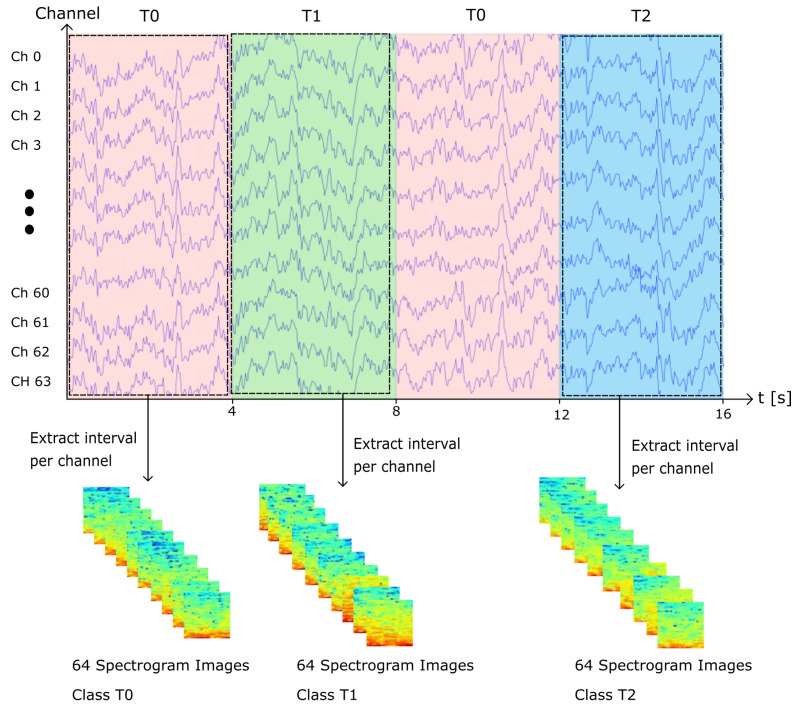
Single-channel spectrogram image generation example. For each class, the interval of time is extracted and transformed into a spectrogram image per channel.

To generate the single-channel spectrogram images, the signal was segmented into time intervals of 4 s. The spectrograms were generated using Python’s API Scipy’s [30] signal function, using a sliding window of 1 s with 90% overlap. The resulting spectrograms were then converted into 32 × 32 RGB images. Therefore, for each channel, approximately 30 spectrograms were generated. In addition, it is important to note that for several recordings, 60 Hz noise can be observed in the spectrogram. In order to evaluate the end-to-end robustness of our models, we decided to not apply any filtering to eliminate noise. Following this method, we generated 604,800 images, from which 302,400 instances belong to class T0 (rest), 152,576 instances belong to class T1 (MI left hand), and 149,824 instances belong to class T2 (MI right hand). An visual example of the previously described method to generate the samples is presented on Figure 4.

#### 2.2.2. Vertically Stacked Spectrograms

**Figure 5:**
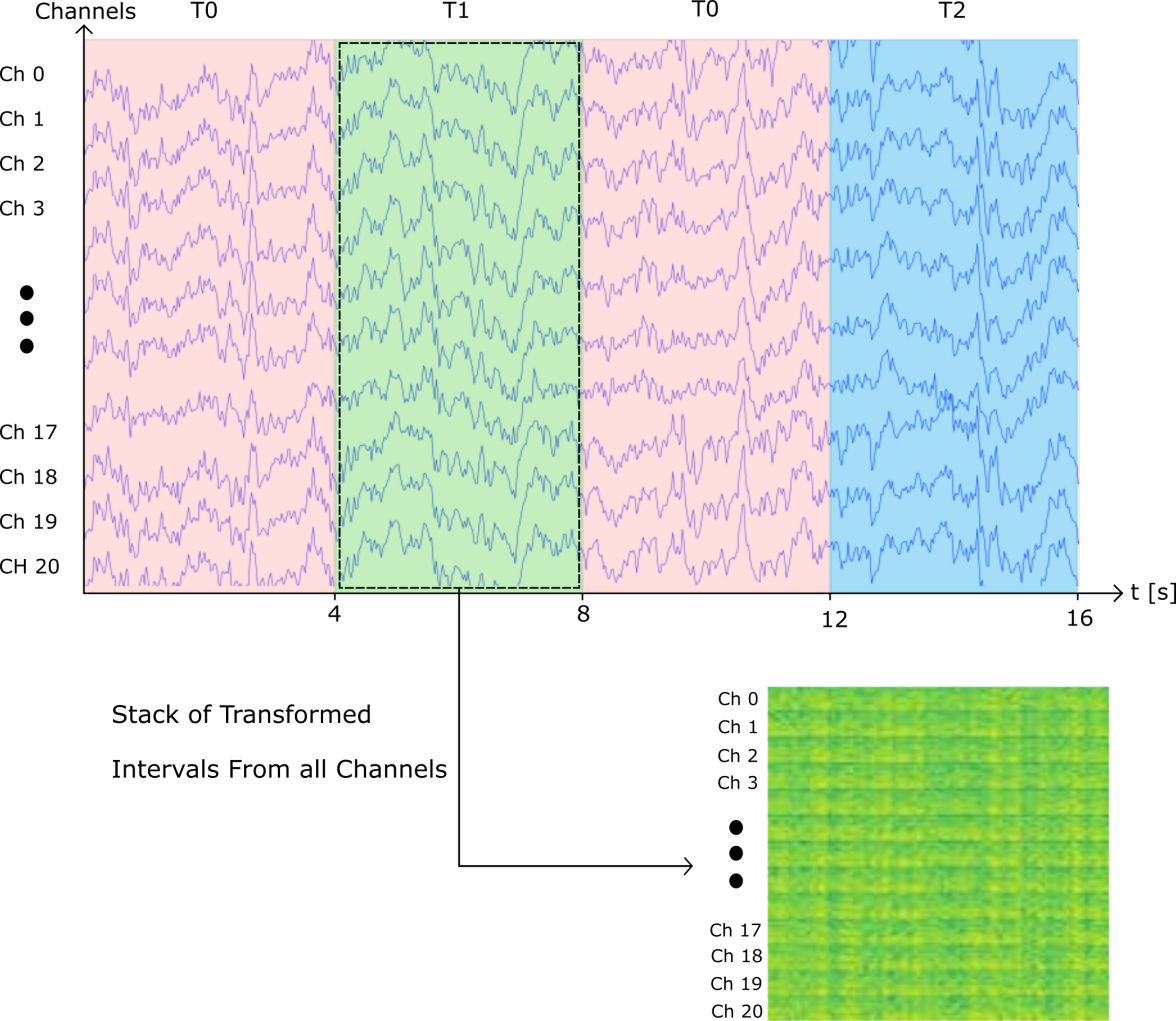
Vertically stacked spectrogram image generation. The transformed signals are stacked over the y-axis to generate the spectrograms from all channels in a single image. Exampled presented with 21-channel selection.

This stage considers an alternative method to generate the spectrograms to represent the data from the EEG signals. The method consists of generating a single image containing the spectrograms of the 64 channels stacked vertically, as illustrated on Figure 5. To create these images, the time interval of 4 s corresponding to a sample is extracted from each channel. The sample is then arranged in a matrix of dimensions 64 by 640. The matrix is then transformed using Python’s API Scipy’s [30] signal function, using a sliding window of 1 s and 90% overlap. The resulting spectrogram image consists of the stacked transform of each channel along the y-axis. In contrast to the previous method to generate spectrograms, this method results in a reduced amount of spectrogram images, since each of the samples is constructed with the 64 channels from the recording, rather than single channels as the previous method. This method generated a total of 9450 sample images, from which 4725 instances belong to class T0 (rest), 2384 instances belong to class T1 (MI left hand), and 2341 instances belong to class T2 (MI right hand).

In addition, variant forms of vertically stacked spectrograms were generated. These images were constructed by reducing the number of electrodes of subjects’ recordings and limiting the information of the recording to channels that correspond to the motor cortex, as seen in Figure 1. As seen in the literature, channels C3, Cz, and C4 contain relevant information in regard to MI. With the aforementioned channel reduction from the EEG recordings, four variants of data representation were designed. Limiting the information to the region of interest of the motor cortex, a 21-, 13-, 9-, and 5-channel selection was devised.

#### 2.2.3. 21-Channel Vertically Stacked Spectrogram

The first variant follows the same procedure for the vertically stacked spectrograms with 64 channels; however, the EEG signal data were limited to the three central channels (C3, Cz, and C4) and their 18 surrounding electrodes that correspond to the region of the cerebral cortex of interest for MI, for a total of 21 channels. The selected channels used for these variants are shown in Figure 1. The channels were reordered so that the central channels C3, Cz, and C4 remained surrounded by their 8 adjacent channels. Therefore, the channels were arranged (according to the visual representation of the 10-10 system seen in Figure 2) from top to bottom, left to right, with the final order of the channels being FC5, C5, CP5, FC3, C3, CP3, FC1, C1, CP1, FCz, Cz, CPz, FC2, C2, CP2, FC4, C4, CP4, FC6, C6, and CP6. Since the variant follows a similar process to the original 64-channel vertically stacked spectrograms, the amount of images generated was the same. With this first variant, a total of 9450 sample images were generated, from which 4725 instances belong to class T0 (rest), 2384 instances belong to class T1 (MI left hand), and 2341 instances belong to class T2 (MI right hand).

#### 2.2.4. 13-Channel Vertically Stacked Spectrograms

**Figure 6:**
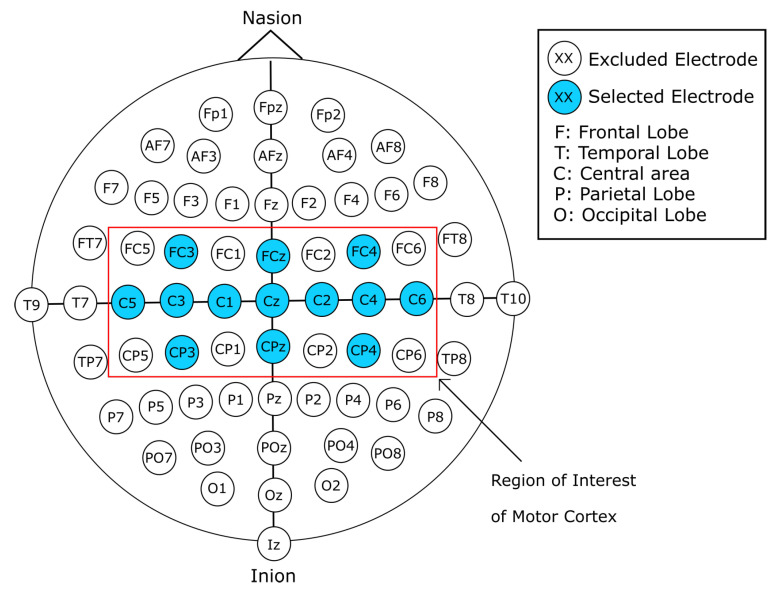
Cross-channel electrodes (blue highlight) employed for the second variant of the vertically stacked spectrograms.

The second variant that spins off the 64-channel vertically stacked spectrograms takes a similar approach as its predecessor. However, the number of channels considered was further reduced to obtain a cross-shaped array of the channels, as shown in Figure 6. The cross-shaped electrode arrangement for this variant takes into consideration only the channels that are horizontally and vertically adjacent to the central channels C3, Cz, and C4. Therefore, the channels FC5, CP5, FC1, CP1, FC2, CP2, FC6, and CP6 were excluded, limiting the data to thirteen channels. To order the channels for the cross-channels vertically stacked spectrograms, the same method as the first variant was used with the exclusion of the aforementioned channels. The order of the channels for these spectrogram images was C5, FC3, C3, CP3, C1, FCz, Cz, CPz, C2, FC4, C4, CP4, and C6. The overall procedure is the same as the previous spectrogram image generation methods, with this approach generating a total of 9450 sample images, where 4725 instances belong to class T0 (rest), 2384 instances belong to class T1 (MI left hand), and 2341 instances belong to class T2 (MI right hand).

#### 2.2.5. 9-Channel Vertically Stacked Spectrograms

Due to the low amount of samples that were generated through the two previously described variants, the third and fourth variant methods were designed to increase the number of samples without the need to increase the number of EEG signal recordings. The third and fourth variants, similar to their predecessors, are based on spatial reduction representation. Therefore, for the third variant the twenty-one channels used for the first variant were subdivided into matrices of nine electrodes. Each of the constructed samples makes use of one of the central electrodes (C3, Cz, and C4), and their eight adjacent electrodes. This allowed us to split the information from the 21-channel vertically stacked spectrogram into three samples containing the information of nine electrodes. The resulting spectrograms share information with each other due to the adjacency positioning of the electrodes. The spectrograms constructed with the C3 electrode as the central channel share the information of electrodes FC1, C1, and CP1 with the spectrograms generated with the Cz electrode as the central channel. Additionally, information from the spectrograms constructed with C4 as the central channel shares the information of electrodes FC2, C2, and CP4 with the spectrograms generated with the Cz electrode as the central channel. This approach allowed to triplicate the number of samples from the first variant method, generating a total of 28,890 sample images, where 14,175 instances belong to class T0 (rest), 7152 instances belong to class T1 (MI left hand), and 7023 instances belong to class T2 (MI right hand).

#### 2.2.6. 5-Channel Vertically Stacked Spectrograms

**Figure 7:**
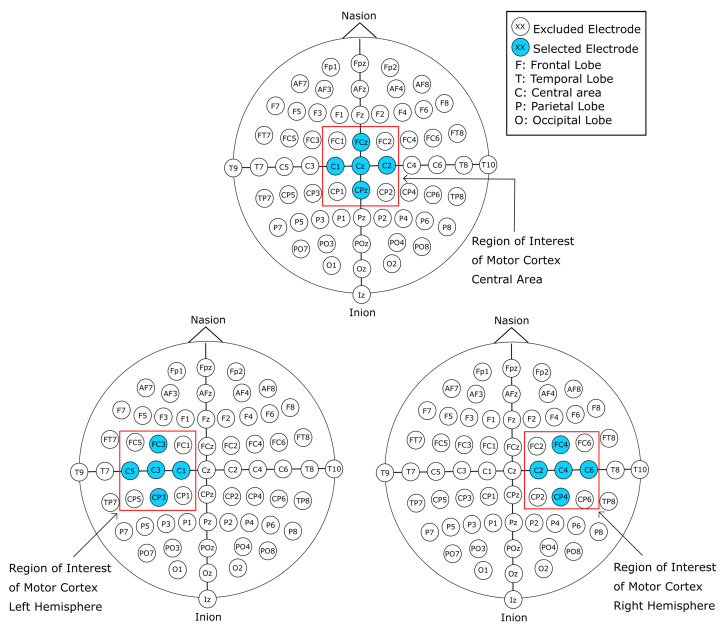
The 5-channel arrangement of electrodes selected for third variant vertically stacked spectrograms images.

As previously stated, the fourth variant follows the same procedure as the third variant with the cross-channel selection as its basis, as shown in Figure 7. The 13-channel cross-channel selection was therefore split into 3 sub arrangements of 5 electrodes, each with one of the central channel electrodes as its center. Similar to the previous variant, some samples share information with each other. The spectrogram images generated with the C3 electrode as the central channel share information with the electrode C1 with the spectrogram created with the Cz electrode as the central channel. In addition, the spectrogram images generated with the C4 electrode as the central channel share information of electrode C2 with the spectrogram images that make use of the Cz electrode as the central channel of the cross-channel spectrogram image. This method allowed to increase the number of samples per class. A total of 28,890 sample images were generated using this method, from which 14,175 instances belong to class T0 (rest), 7152 instances belong to class T1 (MI left hand), and 7023 instances belong to class T2 (MI right hand).

### 2.3. Raw EEG Signal Data Preparation

In addition to the preparation of spectrogram images, the raw EEG signals were prepared in order to serve as an alternative form of input for the CNN models. The preparation of the raw signals followed a similar procedure as the one previously described in Section 2.2. For the raw EEG signal data, ten different approaches were followed, taking into consideration data spatial representation, by reducing the number of electrodes to the area of interest of the motor cortex, as shown in Figure 1. Furthermore, the data were modified so that it would take a representation in the form of volume, similar to that of an RGB image. Finally, single-channel EEG data were prepared to serve as input for one-dimensional CNN models.

#### 2.3.1. 64-Channel Raw EEG Signal Data

Similar to the procedure explained in Section 2.2.2, the raw data for each interval of time of 4 seconds for each class (T0, T1, and T2), were arranged in a matrix of dimensions 64 × 640. No additional processing was performed, and the original order of the data from [27] was preserved. Through this method, a total of 9450 samples were generated. Out of these 9450 samples, 4725 instances belong to class T0 (rest), 2384 instances belong to class T1 (MI left hand), and 2341 instances belong to class T2 (MI right hand).

#### 2.3.2. 21-Channel Raw EEG Signal Data

Following the same steps as in Section 2.2.3, the spatial representation of the original EEG recordings was reduced by limiting the information from the electrodes placed in the motor cortex area shown in Figure 1. Therefore, the raw data for each interval of time corresponding to each class was arranged into a matrix of dimensions 21 × 640. Additionally, the data were reordered in the same manner as in Section 2.2.3, where the central channels C3, Cz, and C4 are surrounded by their adjacent electrodes. The data were therefore arranged from top to bottom, left to right, with the final order of the channels being FC5, C5, CP5, FC3, C3, CP3, FC1, C1, CP1, FCz, Cz, CPz, FC2, C2, CP2, FC4, C4, CP4, FC6, C6, and CP6. Through this method, 9450 samples of data were generated, where 4725 of these instances belong to class T0, 2384 belong to class T1, and 2341 belong to class T2.

#### 2.3.3. 21-Channel Raw EEG Signal: Volume Representation

**Figure 8:**
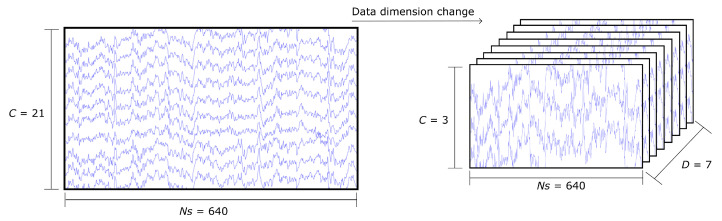
The 21-channel raw EEG signal data preparation for volume representation, where *C* indicates the number of channels, *Ns* indicates the number of samples per channel, and *D* indicates the number of depth layers in the 3D matrix of the volume representation.

Given that a set of stacked arrays does not provide a spatial representation of the EEG channels, the 21-channel samples were re-arranged to mimic the spatial positioning of the EEG electrodes. For that reason, the two-dimensional matrix of 21 × 640 was restructured to a three-dimensional array with dimensions 7 × 3 × 640 as shown in Figure 8, where the first dimension indicates the depth of the matrix, the second dimension the number of channels for each level of depth, and the third dimension represents the length of the time interval. The channels for each level of depth follow the same order as the original two-dimensional matrix; therefore, the first layer contains channels FC5, C5, and CP5, the second layer contains FC3, C3, CP3, and so on. This method created 9450 samples of data, from which 4725 constitute class T0, 2384 are class T1, and 2341 are class T2.

#### 2.3.4. 13-Channel Raw EEG Signal Data

For the 13-channel raw EEG signal data, the process to generate the data samples were similar to the ones described in Section 2.2.4. The cross-shaped channel selection around the central channels C3, Cz, and C4 further reduce the amount of data by excluding electrodes FC5, CP5, FC1, CP1, FC2, CP2, FC6, and CP6. The remaining channels were reordered following the same procedure of top to bottom, left to right, with the final order of the channels being: C5, FC3, C3, CP3, C1, FCz, Cz, CPz, C2, FC4, C4, CP4, and C6. The final shape of the resulting two-dimensional matrix is [13 × 640]. This method created the same amount of data samples as the previous methods, creating a total of 9450 samples of data, 4725 belonging to class T0, 2384 to class T1, and 2341 to class T2.

#### 2.3.5. 13-Channel Raw EEG Signal Data: Volume Representation

**Figure 9:**
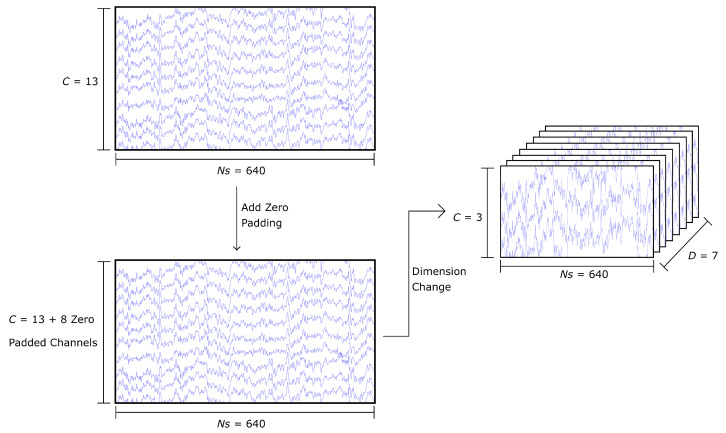
The 13-channel raw EEG signal data preparation for volume representation. Before arranging the signal into a 3D matrix, eight zero-padding arrays are introduced into the sample to be able to generate a 3 × 3 × 3 matrix, where *C* indicates the number of channels, *Ns* indicates the number of samples per channel, and *D* indicates the number of depth layers in the 3D matrix of the volume representation.

Similar to the process of Section 2.3.3, the 13-channel samples were re-arranged to mimic the spatial position of the electrodes. However, due to the missing channels in the data that were excluded to fit the desired arrangement of electrodes, zero-padding had to be added to compensate for the eight missing electrodes for this representation to be transformed into a three-dimensional matrix, as can be observed in Figure 9. With the zero-padding, the data were restructured to have the dimensions of 7 × 3 × 640, following the same procedure described in Section 2.3.3. Through this method, a total of 9450 samples of data were generated, from which 4725 belong to class T0, 2384 belong to class T1, and 2341 belong to class T2.

#### 2.3.6. 9-Channel Raw EEG Signal Data

**Figure 10:**
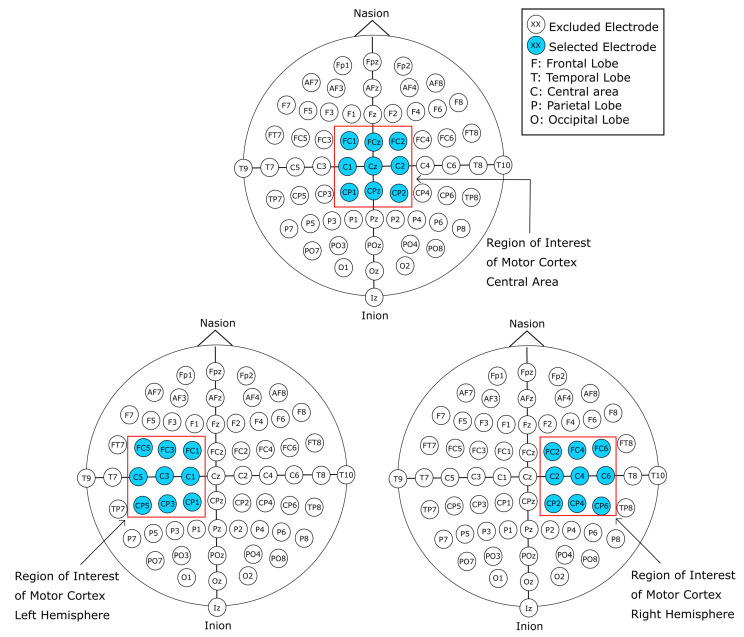
The 9-channel arrangement of electrodes selected for third variant vertically stacked spectrograms images.

To increase the number of samples of raw EEG signals, the 21-channel array was subdivided into three matrices of dimensions 3 × 3 electrodes. The same process was done as in Section 2.2.5, where the two-dimensional array of electrodes was constructed around the central channel electrodes C3, Cz, and C4, as shown in Figure 10. Therefore, the electrodes adjacent to the central channels are used to construct this 9-channel sample of raw data. Additionally, each of the 9-channel matrices were constructed in the same order as previously described methods (top to bottom, left to right). Thus, the first sample is constructed by making use of electrodes FC5, C5, CP5, FC3, C3, CP3, FC1, C1, and CP1; the second sample is constructed by using the electrodes FC1, C1, CP1, FCz, Cz, CPz, FC2, C2, and CP2; the last sample is constructed by using electrode channels FC2, C2, CP2, FC4, C4, CP4, FC6, C6, and CP6. Accordingly, the shape of the data for each of the samples is 9 × 640. This method was employed to increase the number of samples of data for each of the classes that are sought to be classified. The method produced a total of 28,350 samples of data, from which 14,175 belong to class T0, 7152 belong to class T1, and 7023 to class T2.

#### 2.3.7. 9-Channel Raw EEG Signal Data: Volume Representation

The two-dimensional instances of data generated through the 9-channel arrangements of data were reconstructed to create a new form of data representation that seeks to mimic the EEG electrode arrangement observed in Figure 10. For this objective, the data were restructured into three-dimensional matrices with dimensions 3 × 3 × 640. The first dimension describes the depth of the arrangement. The second dimension refers to the number of channels per level of depth, and the last dimension represents the length of the sample interval. Since the only variation for this form of data was the structure in which the data is being represented, the number of total samples is the same as the original set of data from which it was based, resulting in a total of 28,350 samples of data, where 14,175 instances belong to class T0, 7152 belong to class T1, and 7023 to class T2.

#### 2.3.8. 5-Channel Raw EEG Signal Data

Following the same process as in the one described in Section 2.2.6, out of the 21 channels of the highlighted electrodes shown in Figure 1, eight channels (FC5, CP5, FC1, CP1, FC2, CP2, FC6, and CP6) were excluded to reduce the dimensions of the sample. Additionally, the data were separated into sub-arrays of five channels, with the central channels C3, Cz, and C4 at the center of these arrays. The channels are arranged in the same manner as previous sets of data, with a top-to-bottom, left-to-right order. Hence, the order for the first sample makes use of electrodes C5, FC3, C3, CP3, and C1; the second sample is constructed with C1, FCz, Cz, CPz, and C2; finally, the last sample is constructed with electrodes C2, FC4, C4, CP4, and C6. Accordingly, the dimensions of each generated sample are 5 × 640. Through this method we acquired a total of 28,350 samples of data, from which 14,175 instances belong to class T0, 7152 belong to class T1, and 7023 to class T2.

#### 2.3.9. 5-Channel Raw EEG Signal Data: Volume Representation

This form of data representation is the last variant for multi-channel representation of the data. The structure follows the same process as the one described in Section 2.3.7. However, due to the irregular shape of the cross-channel selection for the 5-channel representation, the data were restructured by adding zero-padding to the sections left by the excluded electrodes. With the zero-padding to compensate for the spaces of channels FC5, CP5, FC1, CP1, FC2, CP2, FC6, and CP6, the data were re-arranged into a three-dimensional matrix with dimensions 3 × 3 × 640, where the first dimension refers to the depth of the matrix, the second dimension represents the number of channels per level of depth and the last dimension the length of the sample. The structure of the data takes a similar form as described in Section 2.3.5. With this method a total of 28,350 samples of data were created, where 14,175 instances belong to class T0, 7152 belong to class T1, and 7023 to class T2.

#### 2.3.10. Single-Channel Array of Raw EEG Signal

The last variant form of input considered for this work was single-channel arrays of the raw EEG signal data. The basis of the preparation for this data takes the same steps as the generation of the spectrogram images described in Section 2.2.1, without the transformation of the signal to the frequency domain and the creation of the spectrogram image. These samples of data take the 4 s interval corresponding to a single instance from each channel along with its class label, creating a single vector with a length of 640 (4 s × 160 sample frequency). This final method consists of 604,800 samples of data, from which 302,400 instances belong to class T0, 152,576 instances belong to class T1, and 149,824 instances belong to class T2.

### 2.4. 2D CNN Models

With the RGB spectrogram images as input, three 2D CNN models are proposed in this work for MI 3-class classification. Each convolutional layer of these models is constructed with a convolution filter, a ReLU activation, and a maxpooling, with a fully connected layer to perform the classification. The implementation of these models was carried out in PyTorch [31]. For each model, parameters such as learning rate and learning rate reduction were adjusted by making use of the validation set. The same models were employed for all the multidimensional representations of the EEG signal, with a slight modification at the head of the models to adapt to the different forms of input. For this work, simple architectures were considered in order to maintain a low computational cost. The 2D CNN model architecture allows to analyze the structure of the image-based samples previously described.

#### 2.4.1. Single-Layer 2D-CNN

**Figure 11:**
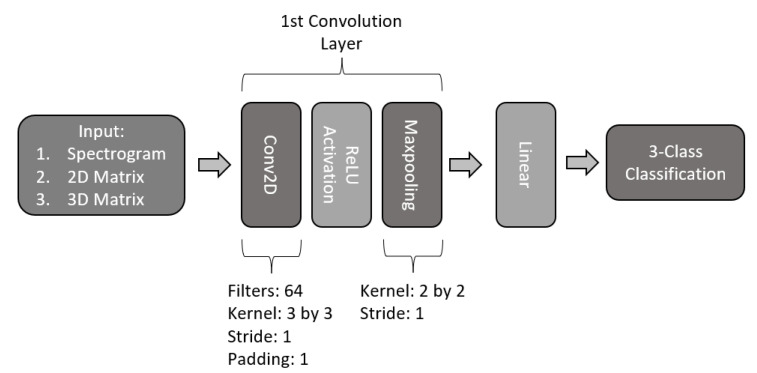
Architecture of single CNN model.

The first proposed model consists of a single-layer CNN, as exemplified in Figure 11. The model is constructed with a 2-dimensional convolution of 64 filters with a kernel size of 3 by 3, stride of 1, and padding of 1. The layer contains a ReLU activation function, followed by a maxpooling filter with a kernel size of 2 by 2 and a stride of 1. The output of this layer is then flattened and is used as input for a fully connected linear output for 3-class classification.

#### 2.4.2. Two-Layered 2D-CNN

**Figure 12:**
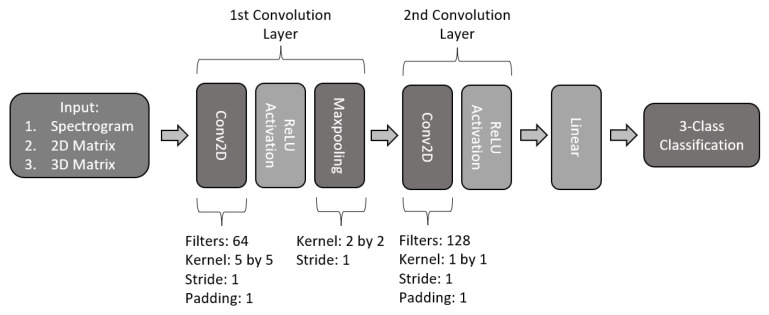
Architecture of two-layered CNN model.

The second proposed model, shown in Figure 12, consists of a two-layered CNN model. Following a similar structure, the first layer is composed of a 2-dimensional convolution of 64 filters with a 5 by 5 kernel, a stride of 1, and a padding of 1, followed by a ReLU activation function and a maxpooling with a kernel 2 by 2 with a stride of 1. For the second layer of this model, a second convolution was constructed with 128 filters with a kernel size of 1, stride of 1, and padding of 1 with a ReLU activation function. Finally, the second layered is connected to a fully connected to perform 3-class classification.

#### 2.4.3. Three-Layered 2D-CNN

**Figure 13:**
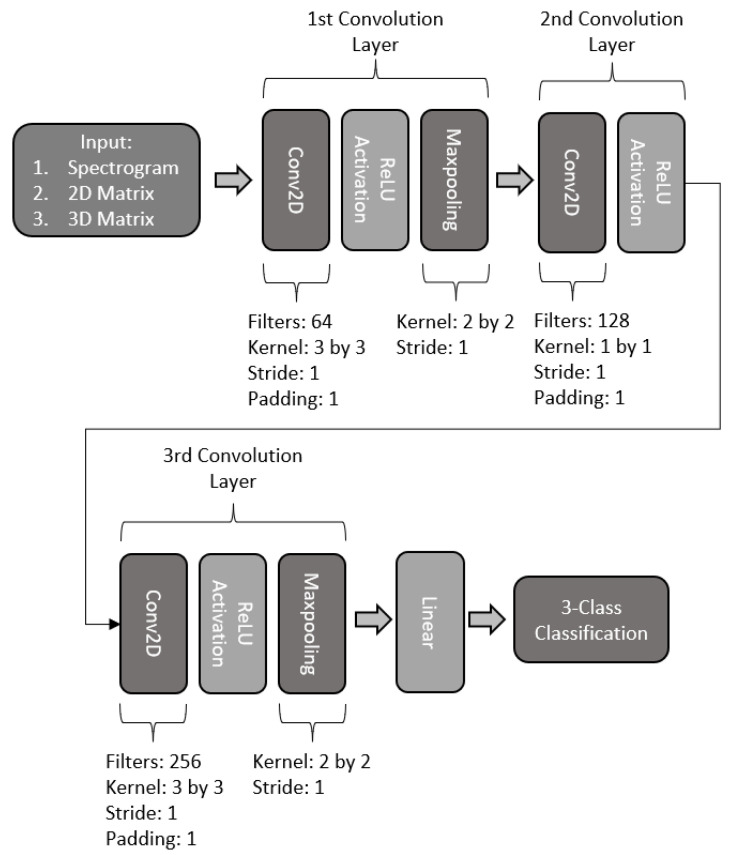
Architecture of three-layered CNN model.

The third proposed model consists of a three-layered model with three convolution layers, with the basic structure shown in Figure 13. The first layer makes use of a 2-dimensional convolution with 64 filters with a kernel size of 3 by 3, a stride of 1, and a padding of 1, followed by a ReLU activation. The convolution is followed by a maxpooling with a kernel size of 2 by 2 and a stride of one. The second layer is constructed using a 2-dimensional convolution with 128 filters, with a ReLU activation. The last convolution layer consists of a 2-dimensional convolution with 256 filters with a kernel of 3 by 3, stride of one, and padding of one with ReLU activation. It is then followed by a maxpooling layer with a kernel of 2 by 2 and a stride of 1. Finally, the output is passed through a fully connected layer to perform the 3-class classification.

#### 2.4.4. Transfer Learning

**Figure 14:**
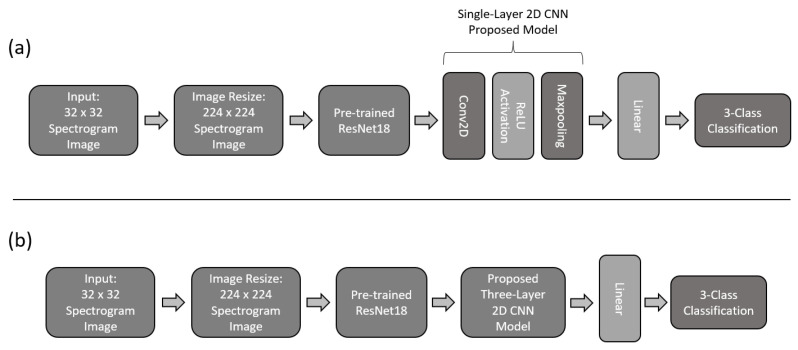
Basic architecture of transfer learning model, using ResNet18’s body. (**a**) Illustrates the implemented model with the devised single-layer models as a tail for the 3-class classification task of MI; (**b**) Illustrates the implemented model with the devised three-layer models as a tail for the 3-class classification task of MI.

For this work, two additional models are proposed by making use of transfer learning. Both models make use of the pre-trained model of ResNet18 [32]. To make use of the pre-trained model, the head of ResNet18 was removed and replaced with our CNN models. For this work, we used the single-layered CNN and three-layared CNN models, as described previously. The basic structure of these models is shown in Figure 14. To fit the spectrogram images into the model, every image had to be resized to a size of 256 × 256 × 3. In order to preserve the features from ResNet18, the body of the model was frozen beforehand and only the head was trained with the training set. After training the complete model, the body was unfrozen and trained for fine-tuning the model using a small learning rate.

### 2.5. 1D CNN Models

As described in Section 2.3.10, the 1D arrays formed through a single-channel time interval of four seconds were used as input for five proposed 1D CNN models. Similar to the 2D CNN models, the 1D model architectures were constructed to have a low computational cost for the MI classification task. Additionally, these models were devised to analyze the raw EEG signal, without any sort of conversion to other domains (e.g., spectrograms). The 1D models allow the analysis of the EEG’s time-domain characteristics.

#### 2.5.1. 1D-CNN

**Figure 15:**
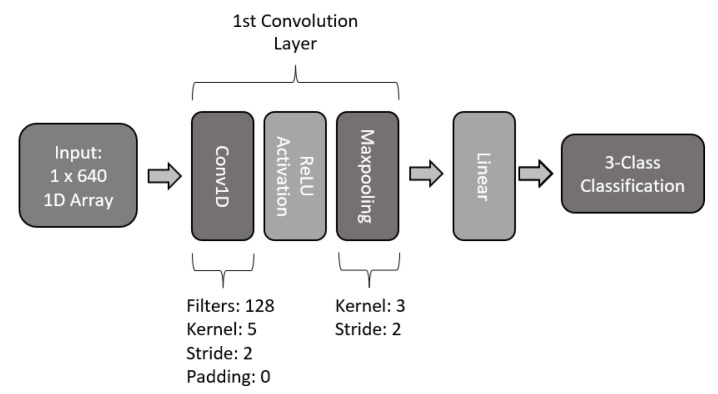
Architecture of single-layer 1D CNN model.

For the first architecture of the proposed models, a single layer was used. The model makes use of a 128 kernel filter with a size of 5, a stride of 2, and no padding. The layer makes use of batch normalization, ReLU activation, and a 1D maxpool of size 3 with a stride of 2. The output of the convolution layer is passed to a fully connected linear network to perform the 3-class classification of MI. The structure of the single-layer 1D CNN model is presented in Figure 15.

#### 2.5.2. Two-Layered 1D-CNN

**Figure 16:**
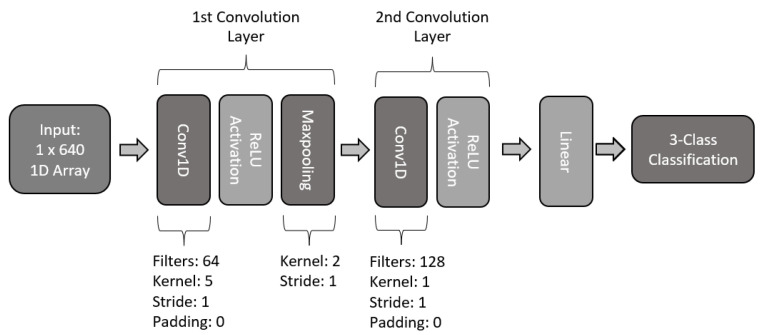
Architecture of two-layer 1D CNN model.

For the second 1D CNN proposed model, an architecture with two layers was designed. The basic architecture is presented in Figure 16. The first layer makes use of 64 kernel filters with a size of size and a stride of 1. This first layer makes use of dropout to reduce overfitting, a ReLU activation, and maxpooling with a kernel size of 2 and a stride of 1. The second layer of this model consists of 128 kernel filters of size 1 and stride of 1, along with ReLU activation. Lastly, the model makes use of a fully connected linear network to perform the 3-class classification.

#### 2.5.3. Three-Layered 1D-CNN

**Figure 17:**
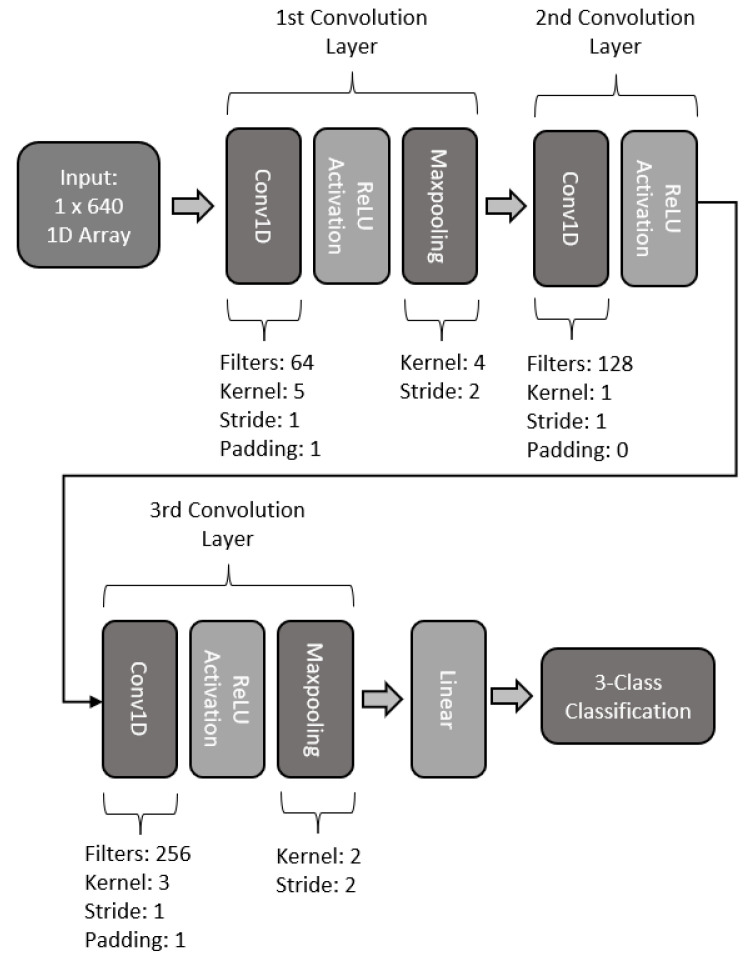
Architecture of three-layer 1D CNN model.

The third proposed 1D CNN model makes use of three convolutional layers. For the first layer a convolution with 64 kernels was used, with a size of 5, stride of 1, and padding of 1. The layer makes use of batch normalization and ReLU activation. The second layer is built upon a convolution with 128 filters, a kernel size of 3, and a stride of 1. The layer makes use of batch normalization and dropout and ReLU activation. The last convolution layer of this model makes use of 256 kernel filters, with a size of 3, stride of 1, and padding of 1. The last layer also includes batch normalization and dropout, along with a ReLU activation function. The basic structure of this model is presented in Figure 17.

#### 2.5.4. Four-Layered 1D-CNN

**Figure 18:**
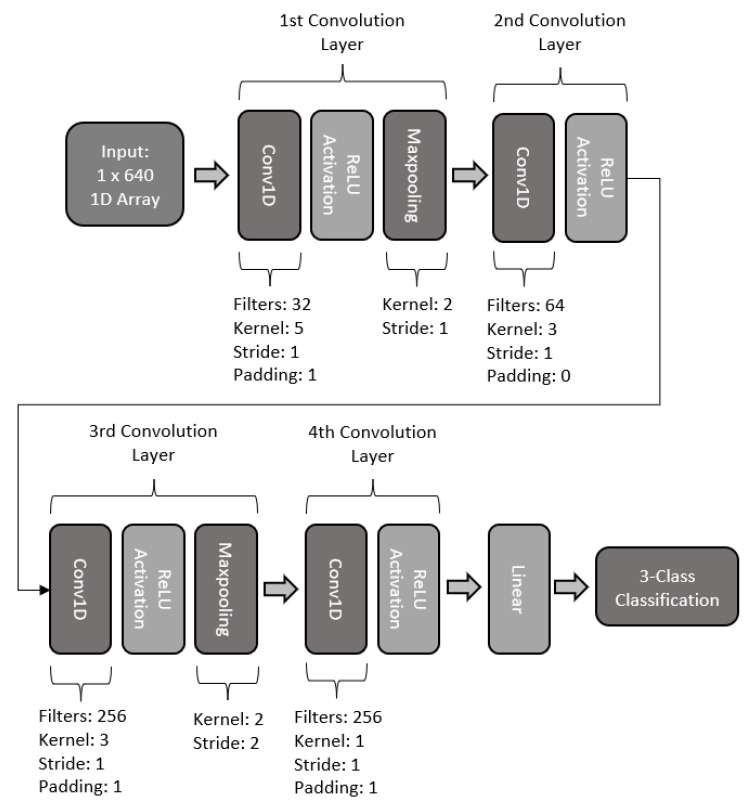
Architecture of four-layer 1D CNN model.

For the fourth model, a 1D CNN with four layers was designed. The basic structure is illustrated in Figure 18 The first convolution layer makes use of 64 filters, with a kernel size of 5, stride of 1, and padding of 1. The layer makes use of batch normalization, ReLU activation, and maxpooling with a kernel size of 2 and a stride of 1. The second layer of the model makes use of 128 filters, with a kernel size of 3 and a stride of 1. This second layer also makes use of batch normalization, dropout, and the ReLU activation function. The third layer of this model consists of 256 kernel filters with a size of 3, stride of 1, and padding of 1. The third also includes batch normalization and dropout, along with ReLU activation and a maxpooling with a kernel size of 2 and stride of 2. The fourth and last layer of this model is constructed with 256 filters, with a kernel size of 1, stride of 1, and padding of 1. The last layer includes a batch normalization and ReLU activation function. Finally, the model makes use of a fully connected linear network for 3-class classification.

#### 2.5.5. Five-Layered 1D-CNN

**Figure 19:**
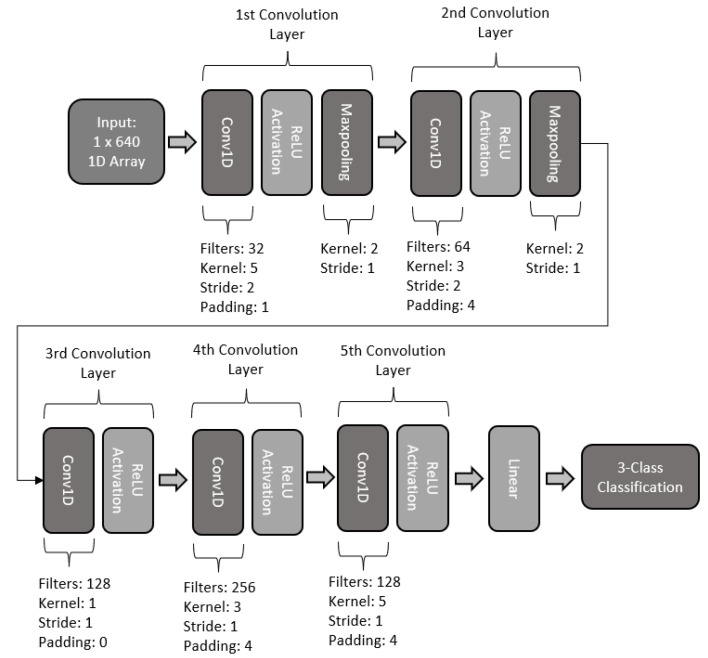
Architecture of five-layer 1D CNN model.

The fifth and last proposed 1D CNN model was designed with 5 convolutional layers. The first layer of the model makes use of a 1D convolution of 32 kernel filters with a size of 5, stride of 2, and padding of 1. The layer makes use of batch normalization, ReLU activation, and a maxpooling with a kernel size of 2 and stride of 1. The second layer convolution makes use of a 64 filter kernel, with a size of 3, stride of 2, and padding of 4. In addition, the layer makes use of batch normalization and dropout, along with ReLU activation, and maxpooling with a kernel size of 2 and stride of 1. The third layer of the model makes use of 128 kernel filters, with a kernel size of 1 and a stride of 1. Additionally, the layer makes use of batch normalization and ReLU activation. For the fourth layer of the model, 256 filters were used, with a kernel size of 3, stride of 1, and a padding of 4. A ReLU activation function along with batch normalization is also included in the fourth layer. The last layer of the proposed model makes use of a reduction of filters, using 128 filters with a kernel size of 5, stride of 1, and padding of 4. The last layer also makes use of batch normalization, ReLU activation, and dropout. Finally, the model makes use of a fully connected linear network to perform 3-class classification. The basic architecture of this model is illustrated in Figure 19.

### 2.6. Hardware and Software

The hardware employed for this work consisted of a personal computer with an AMD Ryzen 3 3200 g processor with a base clock of 3.6 GHz, 16 GB RAM running at 3200 MHz, and an NVIDIA GEFORCE RTX 2060 SUPER with 8 GB VRAM. The models were implemented in *Python*, with the libraries of *numpy* and *pandas* for data manipulation, *scipy* for signal transformation and *Pytorch* for the DL implementation of the proposed CNN models.

## 3. Results and Discussion

This section evaluates and discusses the classification accuracy results on the Physionet Motor Movement/Imagery Dataset’s Task 2 recordings, for three-category classification. Classification accuracy is measured as the ratio of the correctly classified samples over a total number of samples of the test set. For comparison with the state-of-the-art methods in the literature, the results of two studies are reported, both of them presenting a similar paradigm of three-class MI classification using the Physionet public database [27], with different data representation approaches. The first study, presented by Alwasiti et al. [25], proposes a triplet network to classify EEG-based MI tasks. The model makes use of a topographical map of 64 channels constructed with the transformation of the signal to the frequency domain using the Stockwell transform to train the model, then it is tested by feeding triplet pairs, conformed by an anchor, positive and negative labeled epochs, reporting a 65% classification accuracy for three-class MI. The second study, presented by Dose et al. [26], made use of a CNN model for learning generalized features and dimension reduction with a fully connected layer for MI classification. Their work involves a temporal convolution over each of the channels from the 64-channel raw EEG signal, followed by a spatial convolution over the channel axis of the recorded signals. The study reports a global accuracy of 69.82% for the three-class classification of the MI task.

The results and discussion section is divided into four subsections: Section 3.1 addresses the results obtained from the experiments performed with an image-based form of input. Section 3.2 addresses the results obtained from the experiments performed with a 2D matrix representation of the raw EEG signal. Section 3.3 addresses the results obtained from the experiments performed with a 3D matrix representation of the raw EEG signal. Finally, Section 3.4 addresses the experiment performed with the 1D single-channel array of the raw EEG signal.

### 3.1. Results: Image-Based Experiments

**Figure 20:**
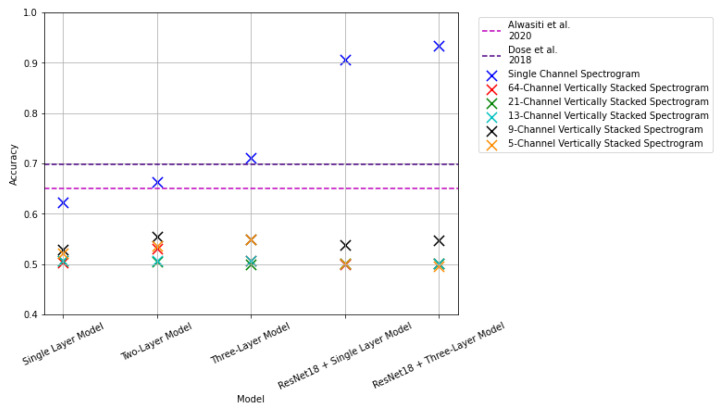
Results of image-based experiments of single-channel spectrogram images and vertically stacked spectrogram images with the 2D CNN models. The pink dashed line indicates results reported in [25]. The purple dashed line indicates results reported in [26].

This subsection presents the results using the proposed CNN models on the image-based representations. The results from these experiments using the test set are summarized in Figure 20 and Table 2.

From Table 2 we can see that our base CNN models with one and two layers have a relatively poor performance when compared to our more complex models, including the one with three convolutional layers and those using transfer learning. Indeed, the three layer model exceeded the results presented in [25] by approximately 6%, and [26] by approximately 2% when using single-channel spectrograms. Furthermore, using this representation, our results based on a pretrained ResNet, i.e., *ResNet18 + Single-Layer CNN* and *ResNet18 + Three-Layer CNN* improved on the previous state of the art significantly, attaining accuracies of 90.55% and 93.32%, respectively.

In contrast, when we evaluate our models using different data representations based on stacking spectrograms, we see that there is a considerable drop in performance from all models. We argue this result is due to the considerable data reduction from generating the samples and the intra-class variability within the same EEG recording, as reported previously [3].

### 3.2. Results: 2D Matrix Raw EEG Signal

**Figure 21:**
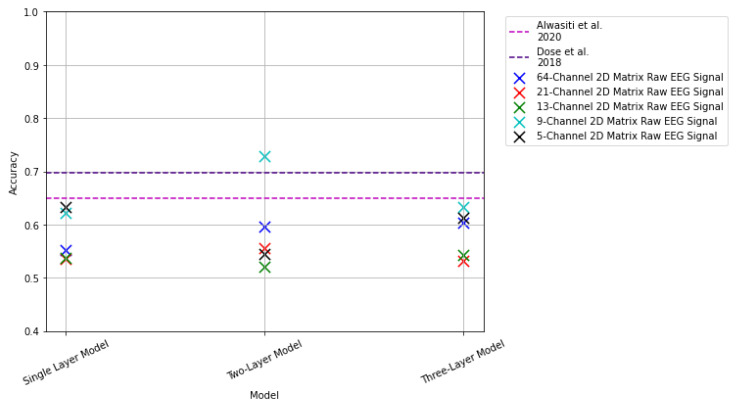
Results of 2D raw EEG signal representation with the 2D CNN models. The pink dashed line indicates results reported in [25]. The purple dashed line indicates results reported in [26].

This subsection presents the results from the CNN models that use the 2D matrix representations as inputs. The results from these experiments are summarized in Figure 21 and in Table 3.

From Table 3, we can see an increase in accuracy in comparison with the vertical stack spectrograms. Hence, we see that the model is able to learn features from a 2D matrix of the raw EEG signal better than from some frequency domain representations. Furthermore, although unable to outperform the state of the art, we observe significant increases in accuracy, particularly the 9- and 5-channel selections as inputs. With these inputs, the single and three-layer model returned accuracies that exceeded 60%. For the two-layer model in combination with the 9-channel 2D signal representation, the model returned a classification accuracy of 72.87%. The model was able to outperform the results presented in [25] by approximately 7% and [26] by 3%.

Even though the combination of the two-layer model with the 9-channel selection samples was able to outperform the state of the art and their spectrogram image counterpart, it was unable to compare favorably against the single-channel spectrograms for the transfer learning implementations.

### 3.3. Results: 3D Matrix Raw EEG Signal

**Figure 22:**
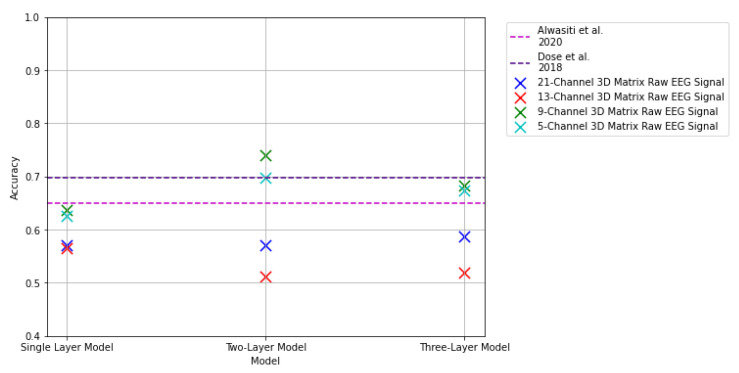
Results of 3D raw EEG signal representation with the 2D CNN models. The pink dashed line indicates results reported in [25]. The purple dashed line indicates results reported in [26].

This subsection presents the results from the proposed CNN models with the 3D matrix representations for the raw EEG signal. The results from these experiments are summarized in Figure 22 and in Table 4. It can be observed that the results from the models with this signal representation returned a significant improvement with similar results to that of the 2D representation. In contrast to their image-based counterpart, the models proved to perform better with the 3D raw EEG signal representation, implying that the models were able to learn features from the 3D matrix representation better than from the spectrograms.

Similar to the results returned through the 2D input presented in Section 3.2, the best results were obtained with the 9- and 5-channel representations, returning accuracies that exceeded a 60% classification accuracy performance. These results imply that focusing on the information around the central channels conveys better features for our models, rather than taking information from the complete set of recordings. The best results were obtained with the two-layer model CNN model. The two-layer model with the 9-channel selection serving as input returned a classification accuracy that outperformed the results presented by [25] by 8%, and [26] by approximately 5%. The same model with a 5-channel selection as input of the 3D representation of the signal was able to outperform the results presented by [25] by 4%, and returning a classification accuracy on par with the results of [26], with approximately a 0.02% difference in the accuracy.

### 3.4. Results: 1D Raw Signal

**Figure 23:**
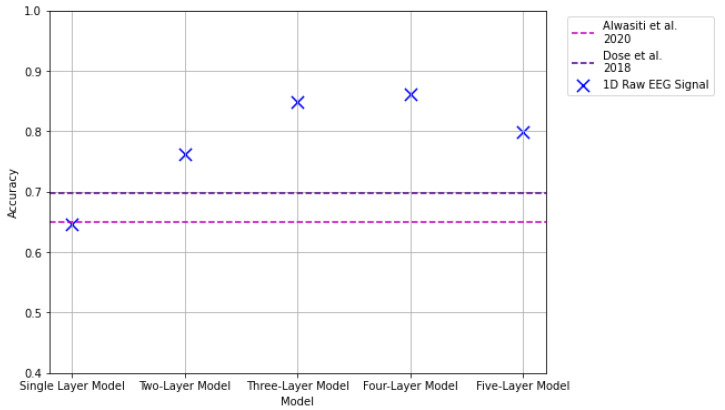
Results of 1D raw EEG signal representation with the 1D CNN models. The pink dashed line indicates results reported in [25]. The purple dashed line indicates results reported in [26].

The last input modality considered in this work consists of 1D arrays from the raw EEG signal. Each data sample represents an interval of time in which a labeled task was executed. For this input format, five models were proposed. The results from these experiments are summarized and compared against the state of the art in Figure 23 and Table 5.

The single-layer 1D CNN model returned a classification accuracy of 64.69%. The second proposed model, consisting of a two-layer 1D CNN model, returned a classification accuracy of 76.29%; with the three-layer 1D CNN model returning 84.87% classification accuracy. The four-layered model outperformed the previous models, with a classification accuracy of 86.12%. Lastly, the five-layered 1D CNN returned an accuracy of 79.58%, leading to better accuracies than those obtained using 2 and 3D data representations. Additionally, we can see a significant improvement in the vertically stacked spectrogram representations of the signal. This implies that the information conveyed by the individual channels helps the feature extraction process that represents the MI task. In contrast to the state of the art, the result from the proposed single-layer model was not able to outperform the reported results from [25,26], with the obtained classification accuracy being approximately 0.5% and 5% lower than those reported in their studies respectively. However, the accuracies obtained from the other four models were able to outperform their reported results. It is also interesting to note that although the results from these models are not as good as those from the single-channel spectrogram images presented in Section 3.1, with a difference of approximately a 7% difference between the proposed four-layered 1D CNN model and the *ResNet18 + Three Layer CNN* transfer learning implementation, there is a significant reduction in computational costs since the time required to prepare 1D arrays is considerably less than that required to generate the spectrograms.

## 4. Conclusions

EEG signals present a 1D representation of the physiological activity of mental tasks. The recordings obtained from EEGs show excellent time-domain granularity, sacrificing spatial resolution. To overcome this obstacle, different forms of data representation can be designed to mimic the spatial representation of the actual physical signals. In this work, an extensive experimentation over different forms of EEG data representations was explored. Data representations included the generation of spectrogram images from single channels in a single image, and multiple channels within a single data sample. In addition, the raw signal was rearranged into 2D and 3D matrices of the EEG’s most significant channels. The best accuracy performances were observed with the single-channel spectrogram representation with a transfer learning using a pre-trained ResNet18 in combination with a three-layered CNN model for MI classification, with an accuracy of 93.32%. As can be observed on Table 6, high accuracy performance was also obtained using 1D CNN models, indicating that raw channel information represents a viable form of input to extract features suitable for MI classification, with the fourth model having the highest performance of 86.12%. Given their relative simplicity compared to 2D CNN models, further experimentation is needed to validate the real-time applications of these 1D CNN models. Additionally, alternate forms of the EEG signal representation also provided results that proved to be viable options for the classification of MI, e.g., the two-layered model with a volume representation of the signal that returned a classification accuracy of 73.93%. Due to its nature, the physiological signal can be complex, and relevant features within the signal may vary among the subjects, hence the correct input format is relevant to achieve high accuracies. The main contributions brought by this project are summarized as follows:

- Extensive experimentation with different EEG representation modalities using single-channel spectrograms, 3D raw EEG signal, and 1D raw EEG signal analysis.
- Novel deep learning algorithms are designed specifically for each of the data representation modalities.
- Analysis of the selection of distinct EEG channels for optimal MI representation.

Finally, we consider it relevant to point out that the 9- and 5-channel EEG representations are highly important. Although channel selection is a common method employed for EEG analysis, the selection is usually performed over a complete region of interest with a symmetrical pairing of electrodes from both hemispheres. The presented 9- and 5-channel selection process consists of the segmentation of the recording into samples that represent different areas of the motor cortex, independently of a symmetrical pairing among hemispheres. Furthermore, through this data preparation, additional samples representing the MI task were able to be generated. Although the results obtained with these forms of the EEG signals’ representation were not the highest, the results were able to provide an accuracy performance higher than that reported previously in the state of the art, and should be explored further in future endeavours.

## 5. Future Work

The end-line of this work would seek a real-time application. This work could be implemented for rehabilitation routines for patients with a form of motor skill impairment. Therefore, future work would involve the improvement of the analysis of the 1D array as well as the 2D and 3D matrix representation, since these formats entail the lowest computational resources. Additionally, the implemented models used to analyze the 1D, 2D, and 3D representations provided promising results, and further exploration of these representations of the EEG signal could lead to viable applications for an online experiment. After improving the models, the following step would be to test the models to analyze EEG signals of previously unseen data.

Likewise, given their ability to abstract information, our models could be used in other research/engineering problems based on EEG signals. For example, they could be used as pre-trained models used in epilepsy, sleep, and ADHD, just to name a few. This way, it would be possible to use transfer learning for EEG analysis.

## Figures and Tables

**Table 1:** Contents of each recording from Physionet’s EEG Motor Movement/Imagery Dataset [27].

Recording 01	Baseline recording with open eyes
Recording 02	Baseline recording with closed eyes
Recording 03	Task 1: Motor execution of one hand (left or right)
Recording 04	Task 2: Motor imagery of one hand (left or right)
Recording 05	Task 3: Motor execution of both hands or both feet
Recording 06	Task 4: Motor imagery of both hands or both feet
Recording 07	Task 1
Recording 08	Task 2
Recording 09	Task 3
Recording 10	Task 4
Recording 11	Task 1
Recording 12	Task 2
Recording 13	Task 3
Recording 14	Task 4

**Table 2:** Image-based inputs results. The 1-, 2-, and 3-layer simple CNN models returned a test accuracy of 62.26%, 66.38%, and 71.08%, respectively. With the transfer learning implementation, the models returned a test accuracy of 90.55% and 93.32%. In contrast, using vertically stacked spectrograms resulted in a lower classification accuracy, unable to outperform the state of the art. Results that outperformed both studies from the state of the art are in bold.

Model	Input	Accuracy [%]
[25]	Topographical Map	65.00%
[26]	2D Raw EEG signal	69.82%
Single-Layer CNN	Single-Channel Spectrogram Image	62.26%
	64-Channel Vertically Stacked Spectrogram Image	50.37%
	21-Channel Vertically Stacked Spectrogram Image	50.68%
	13-Channel Vertically Stacked Spectrogram Image	50.68%
	9-Channel Vertically Stacked Spectrogram Image	52.92%
	5-Channel Vertically Stacked Spectrogram Image	52.16%
Two-Layer CNN	Single-Channel Spectrogram Image	66.38%
	64-Channel Vertically Stacked Spectrogram Image	53.015%
	21-Channel Vertically Stacked Spectrogram Image	50.68%
	13-Channel Vertically Stacked Spectrogram Image	56.68%
	9-Channel Vertically Stacked Spectrogram Image	55.55%
	5-Channel Vertically Stacked Spectrogram Image	53.61%
**Three-Layer CNN**	**Single-Channel Spectrogram Image**	**71.08%**
	64-Channel Vertically Stacked Spectrogram Image	50.68%
	21-Channel Vertically Stacked Spectrogram Image	50.05%
	13-Channel Vertically Stacked Spectrogram Image	56.68%
	9-Channel Vertically Stacked Spectrogram Image	54.92%
	5-Channel Vertically Stacked Spectrogram Image	55.02%
**ResNet18 + ** **Single-Layer CNN**	**Single-Channel Spectrogram Image**	**90.55%**
	64-Channel Vertically Stacked Spectrogram Image	49.94%
	21-Channel Vertically Stacked Spectrogram Image	50.15%
	13-Channel Vertically Stacked Spectrogram Image	50.15%
	9-Channel Vertically Stacked Spectrogram Image	53.86%
	5-Channel Vertically Stacked Spectrogram Image	50.19%
**ResNet18 +** **Three-Layer CNN**	**Single-Channel Spectrogram Image**	**93.32%**
	64-Channel Vertically Stacked Spectrogram Image	50.15%
	21-Channel Vertically Stacked Spectrogram Image	50.15%
	13-Channel Vertically Stacked Spectrogram Image	50.15%
	9-Channel Vertically Stacked Spectrogram Image	54.70%
	5-Channel Vertically Stacked Spectrogram Image	49.59%

**Table 3:** The 2D raw EEG signal for 2D CNN models results. In contrast to the image-based input, a slight increase in performance can be observed in accuracy. Most of the devised representations were able to achieve performances higher than 50%. The best results obtained with this form of input representation were obtained with the 2D raw 9- and 5-channel EEG signal sample, with an accuracy of 62.22% and 63.31% in combination with the single-layer CNN model. The three-layer CNN model with the 9- and 5-channel selections was able to reach a classification accuracy of 63.28% and 61.26% respectively. In the case of the two-layer CNN model in combination with the 64-channel selection, it was able to return a classification accuracy of 59.68%. Lastly, the only one that was able to achieve a result that outperformed the state of the art was that of the two-layer CNN model in combination with the 9-channel selection of the 2D raw EEG signal, with a classification accuracy of 72.87%. Results that outperform the state of the art are highlighted in bold.

Model	Input	Accuracy [%]
[25]	Topographical Map	65.00%
[26]	2D Raw EEG signal	69.82%
Single-Layer CNN	2D Raw 64-Channel EEG Signal	55.23%
	2D Raw 21-Channel EEG Signal	53.54%
	2D Raw 13-Channel EEG Signal	53.75%
	2D Raw 9-Channel EEG Signal	62.22%
	2D Raw 5-Channel EEG Signal	63.31%
**Two-Layer CNN**	2D Raw 64-Channel EEG Signal	59.68%
	2D Raw 21-Channel EEG Signal	55.66%
	2D Raw 13-Channel EEG Signal	52.16%
	**2D Raw 9-Channel EEG Signal**	**72.87%**
	2D Raw 5-Channel EEG Signal	54.39%
Three-Layer CNN	2D Raw 64-Channel EEG Signal	60.42%
	2D Raw 21-Channel EEG Signal	53.12%
	2D Raw 13-Channel EEG Signal	54.25%
	2D Raw 9-Channel EEG Signal	63.28%
	2D Raw 5-Channel EEG Signal	61.26%

**Table 4:** Raw EEG signal as volume representation results. It can be observed that the results show a significant improvement in comparison to those from the vertically stacked spectrograms. Similar to the 2D raw EEG inputs, the best results are observed within the 9- and 5-channel selections. Through this EEG representation, the single-layer CNN model returned a classification accuracy of 63.70% and 62.64% with the 9- and 5-channel selections respectively. For the two-layer CNN model, 9- and 5-channel selections delivered results that outperformed the state of the art, with a classification accuracy of 73.93% for the 9-channel selection and 69.84% for the 5-channel selection. Lastly, the three-layer CNN model was only able to outperform the results presented by [25], with an accuracy of 68.35% and 67.37% for the 9- and 5-channel selection respectively. Best results that outperformed the state of the art are highlighted in bold.

Model	Input	Accuracy [%]
[25]	Topographical Map	65%
[26]	2D Raw EEG signal	69.82%
Single-Layer CNN	3D Raw 21-Channel EEG Signal	57.14%
	3D Raw 13-Channel EEG Signal	56.50%
	3D Raw 9-Channel EEG Signal	63.70%
	3D Raw 5-Channel EEG Signal	62.64%
**Two-Layer CNN**	3D Raw 21-Channel EEG Signal	57.14%
	3D Raw 13-Channel EEG Signal	51.21%
	**3D Raw 9-Channel EEG Signal**	**73.93%**
	**3D Raw 5-Channel EEG Signal**	**69.84%**
Three-Layer CNN	3D Raw 21-Channel EEG Signal	58.73%
	3D Raw 13-Channel EEG Signal	51.85%
	3D Raw 9-Channel EEG Signal	68.35%
	3D Raw 5-Channel EEG Signal	67.37%

**Table 5:** The 1D raw EEG signal array results. Contrary to the previous forms of data representations, models based on the 1D array achieved significant improvements in the classification of MI in comparison with the previous experiments. Although the first 1D CNN model was unable to outperform the studies from the state of the art, the rest of the models returned results with a significant increase in accuracy performance. The best results were obtained through the three- and four-layered models, with an accuracy of 84.87% and 86.12% respectively. Best results that outperformed the state of the art are highlighted in bold.

Model	Input	Accuracy [%]
[25]	Topographical Map	65%
[26]	2D Raw EEG signal	69.82%
Single-Layer 1D CNN	1D Raw EEG Signal Array	64.69%
**Two-Layer 1D CNN**	**1D Raw EEG Signal Array**	**76.29%**
**Three-Layer 1D CNN**	**1D Raw EEG Signal Array**	**84.87%**
**Four-Layer 1D CNN**	**1D Raw EEG Signal Array**	**86.12%**
**Five-Layer 1D CNN**	**1D Raw EEG Signal Array**	**79.58%**

**Table 6:** Comparison of best results presented in this work in comparison with the studies from the state of the art. The three best results for each of the proposed models are presented. It can be observed that the highest accuracy scores were obtained through the single-channel spectrogram images as input for the transfer learning implementations, with scores higher than 90%. Nevertheless, promising results were obtained through the 1D representations of the signal with the 1D CNN models, with accuracies that were able to exceed an 80% accuracy performance. In contrast with the single-channel spectrogram images, these samples required significantly less time to prepare. Additionally, the 9- and 5-channel 2D and 3D representations of the signals provided promising results with the CNN models, with results that were able to outperform the state of the art. Further exploration of these forms of representations as input for DL models might yield promising results for the MI classification task. The best five results are highlighted with bold.

Model	Input	Accuracy [%]
[25]	Topographical Map	65%
[26]	2D Raw EEG signal	69.82%
Single-Layer CNN	2D Raw 5-Channel EEG Signa	63.31%
	3D Raw 9-Channel EEG Signal	63.70%
	3D Raw 5-Channel EEG Signal	62.64%
Two-Layer CNN	2D Raw 9-Channel EEG Signal	72.87%
	3D Raw 9-Channel EEG Signal	73.93%
	3D Raw 5-Channel EEG Signal	69.84%
Three-Layer CNN	Single-Channel Spectrogram Image	71.08%
	3D Raw 9-Channel EEG Signal	68.35%
	3D Raw 5-Channel EEG Signal	67.37%
**ResNet18 + ** ** Single-Layer CNN**	**Single-Channel Spectrogram Image**	**90.55%**
	9-Channel Vertically Stacked Spectrogram Image	53.68%
	5-Channel Vertically Stacked Spectrogram Image	50.19%
**ResNet18 + ** ** Three-Layer CNN**	**Single-Channel Spectrogram Image**	**93.32%**
	9-Channel Vertically Stacked Spectrogram Image	54.70%
	64-Channel Vertically Stacked Spectrogram Image	50.15%
Single-Layer 1D CNN	1D Raw EEG Signal Array	64.69%
Two-Layer 1D CNN	1D Raw EEG Signal Array	76.29%
**Three-Layer 1D CNN**	**1D Raw EEG Signal Array**	**84.87%**
**Four-Layer 1D CNN**	**1D Raw EEG Signal Array**	**86.12%**
**Five-Layer 1D CNN**	**1D Raw EEG Signal Array**	**79.58%**

## Data Availability

A publicly available dataset was analyzed in this study. This data can be found here: https://doi.org/10.13026/C28G6P (accessed on 10 July 2022).
